# Benefits Trafficking: human trafficking of older adults and adults with disabilities

**DOI:** 10.3389/fresc.2023.1305926

**Published:** 2024-01-22

**Authors:** Anna Thomas, Heather Strickland

**Affiliations:** ^1^ Department of Human Services, Division of Aging Services, Georgia Bureau of Investigation Crimes Against Disabled and Elderly Task Force, Atlanta, GA, United States; ^2^ Georgia Bureau of Investigation Crimes Against Disabled and Elderly Task Force, Atlanta, GA, United States

**Keywords:** Benefits Trafficking, unlicensed personal care home, at-risk adults, financial exploitation, Georgia Bureau of Investigation

## Abstract

An emerging type of trafficking is targeting overlooked at-risk adults. Benefits Trafficking is the systematic recruitment, harboring, neglect, and financial exploitation of elder and disabled adults who receive government benefits such as Social Security, Veteran's Benefits, Medicaid, and Medicare. Traffickers often pose as kind-hearted individuals offering to provide care for at-risk adults in an in-home setting. Once recruited, at-risk adults are stripped of their government benefits, held against their will, moved from location to location, and denied basic needs such as food, clothing, and adequate shelter. While Benefits Trafficking is a basic civil rights violation issue, it is also a growing public health issue. Victims of Benefits Trafficking are often the forgotten at-risk adults who have fallen through the cracks of various mental health systems, are unhoused, and no longer have a social support system in place. This area of human trafficking is unresearched and its prevalence is largely unknown outside of the few entities working in this space. This paper focuses mainly on raising awareness of Benefits Trafficking and suggestions for future funding and research initiatives.

## Introduction

### At-risk adults

At-risk Adults and Vulnerable Adults are terms used to collectively refer to older adults and disabled adults. Each state defines the age of an elder differently and some states do not have a disabled adult in their protected class. Official Code of Georgia Annotated (O.C.G.A.) §16-5-100, defines an elder as a person who is 65 years of age or older and a disabled adult as a person aged 18 or older who has an incapacitation that is physical or mental (such as Dementia and Alzheimer's) [(1) amended].

According to the United States Census Bureau, “by 2060, nearly one in four Americans is projected to be an older adult,” making up about 23% of the population (2). The Administration for Community Living noted in 2020 that 16% of the U.S. population, or one in every seven Americans, is 65 or older (3). Georgia has over 1 million people aged 65+, making up almost 15% of Georgia's population. In the last ten years (2010–2020), Georgia has seen over a 50% increase in people aged 65+ (4).

It is difficult to calculate the percentage of disabled adults that fit the definition of Georgia's statute and most states for that matter. The most current United States Census reported that 12.6 percent of Georgia's population has a disability (4).

## Personal care homes

Georgia law, O.C.G.A. § 31-7-12, defines a personal care home as “any dwelling, whether operated for profit or not, which undertakes through its ownership or management to provide or arrange for the provision of housing, food service, and one or more personal services for two or more adults who are not related to the owner or administrator by blood or marriage” [(5) amended]. Personal services include activities of daily living, bathing, dressing, eating, and taking medication. Other terms used to describe these homes around the United States are board-and-care and/or group homes. Personal care homes are required to be licensed in Georgia by Healthcare Facility Regulation Division (HFRD). HFRD licenses and inspects personal care homes and nursing homes, and conducts non-criminal investigations of reports of abuse, neglect, and exploitation of at-risk adults who are in these facilities and not in private homes. If an owner/operator has multiple addresses where they are operating a personal care home, each address is required to be licensed by HFRD. Healthcare Facility Regulation Division investigations of abuse, neglect, and exploitation (ANE) that are determined to be criminal in nature (i.e., substantiated) would be forwarded to local law enforcement. Internal qualitative research indicates that some states do not have an agency that oversees or licenses personal care homes. These states have noted rampant exploitation and neglect in these homes.

The 1999 ruling of the U.S. Supreme Court in Olmstead v. L.C. created a need for licensed personal care homes in the community [(6), 2]. Vulnerable adults with limited financial resources were looking for care in community settings. Unfortunately, demand quickly overtook supply and greedy providers began to warehouse vulnerable adults into clandestine homes. Due to systemic issues such as the lack of mental health services and affordable, safe housing, unlicensed homes fill a legitimate need with malicious and illegitimate means. Compounding variables contribute to Benefits Trafficking victimization. Social determinants of health play a key role in identifying who is suspectable to this type of trafficking and why. Investigations reveal that many victims are unhoused due to unemployment, food insecure despite receiving government benefits, lack transportation to and from adequate health-related services including mental health services, and are socially isolated from any sort of support system such as family or friends. Many times, victims are funneled into a trafficker's unlicensed home through a faith community outreach organization, after discharge from a mental health facility or hospital, through placement by community behavioral health services, or by public guardians and Adult Protective Services (APS).

Benefits Trafficking often co-occurs within the operation of unlicensed personal care homes. The at-risk adult and vulnerable adult population is recruited by a trafficker who has one licensed care home, and multiple other homes that are unlicensed which are then operated by the family of the trafficker (7). Due to the licensing and regulation requirements, it is often more profitable to operate an unlicensed care home. In past criminal investigations, traffickers would bring in new residents from jails or hospitals and observe them for a few days. After this, the trafficker would move the victim to a hidden home in which the victim would be a better fit. This would in turn open another bed in their licensed home to accept another referral. Images 1 and 2 in the Supplemental Material link illustrate examples of unlicensed personal care homes.

Traffickers warehouse at-risk adults for the purpose of obtaining the victims' benefits (SNAP-Food Stamps, Veterans Administration benefits, and Social Security Disability or Supplemental Security Income). Residents may be moved from one unlicensed care home to another to avoid detection by both law enforcement and the licensing regulatory authorities. Through on-site data collection, certain patterns have emerged in many unlicensed personal care homes (Figure 2). Additionally, it is rumored but with limited intelligence data about traffickers selling residents to other owners and operators of unlicensed personal care homes. If an owner/operator can recruit a victim who receives a larger monthly benefit amount, the trafficker will “sell” the victim to another unlicensed care home for a partial monthly amount. Law enforcement currently does not have the opportunity or resources to track down these types of claims in their investigations. However, during interviews, victims advised in general terms that they were moved from home to home with different operators. See Figure 2 for unlicensed personal care home red flags.

## Prevalence

Approximately 23 states have identified that Benefits Trafficking is also occurring in their state. Other states may classify the crime in other ways such as theft, financial exploitation, neglect, and abuse of both older adults citizens and disabled adults.

A federal indictment brought in Pennsylvania charged Linda Weston and four co-defendants with labor and sex trafficking. The conspirators held six adults with cognitive disabilities in forced labor and sexual servitude for years, forcing some of the victims to have children. Weston targeted individuals with disabilities who were estranged from their families, convincing the victims to move into Weston's home. Once the victims moved in, Weston became the “representative payee” for each, stealing her victims' Social Security benefits. Weston and her co-conspirators kept the captives locked in closets, cabinets, basements, and attics. The traffickers trafficked two female victims into forced commercial sex. The traffickers subjected the victims to extreme physical and sexual abuse. Two victims died in captivity. (United States v. Weston, No. 13-025-1; E.D. Pa. Jan. 22, 2013). Weston pled guilty to all charges. She was sentenced to life in prison plus 80 years and ordered to pay the Social Security Administration over $270,000 (8).

There exists a connection between victims of Benefits Trafficking and prior involvement with APS. Data snapshots in 2017 and 2018 showed approximately 39.8% of at-risk adults who had been relocated from an unlicensed personal care home were persons 65 years of age and older who were vulnerable because of age and mental/cognitive/physical decline. Research indicated that formerly relocated at-risk adult victims’ names also appeared in referrals for unlicensed personal care homes and as part of APS intake reports for allegations of ANE. To illustrate the significance of this issue, approximately 15% of the at-risk adults who had been relocated from an unlicensed personal care home had subsequently found themselves residing in another unlicensed personal care home. In 2017, approximately 40% of all relocated victims had an APS history, with 64.6% of the victims having one previous allegation of ANE. Further broken down, 37.6% of those relocated from unlicensed or licensed personal care homes and 37.2% of those relocated from personal homes had been clients of prior APS investigations and/or interventions. Out of those who had previous APS reports of ANE by an unlicensed personal care home provider, 14.4% were still with or ended up back with the same trafficker after relocation occurred. These findings demonstrate the lasting endangerment associated with having an APS history and being victimized by a trafficker in an unlicensed personal care home. Data showed that 54.6% of emergency relocations from an unlicensed personal care home also prompted co-occurring APS investigations. Current State Fiscal Year numbers show an increase in the number of individuals relocated from unlicensed personal care homes from 18 individuals in 2022 to 25 individuals in 2023 (9). Roughly 70% of emergency relocation cases include law enforcement involvement, and of those, approximately 30% were initiated by law enforcement (10).

## Abuse, neglect, and financial exploitation

Georgia has specific statutes that address abuse, neglect, and financial exploitation crimes committed against at-risk adults. Additionally, when referring to at-risk adult abuse, the abuse component encapsulates any multitude of crimes including but not limited to physical abuse, neglect, financial exploitation, misuse of power of attorney, benefits fraud, undue influence, and Benefits Trafficking. Reports of ANE are steadily increasing. In State Fiscal Year 2022, Georgia's APS program documented 26,254 total reports and 28,921 allegations of ANE (9).

Older adults and disabled adults are considered one of the most vulnerable populations. They are preyed upon as attractive targets by perpetrators due to at-risk adults' incapacitations which, in turn, make some of them unable to defend or advocate for themselves. In addition, the amount of assets this population may have accumulated such as Social Security benefits, food stamps, retirement, and stimulus checks also makes them attractive targets. Our research found an overlapping component of mental health conditions co-occurring with disabilities and age with victims of Benefits Trafficking.

**Figure 1:**
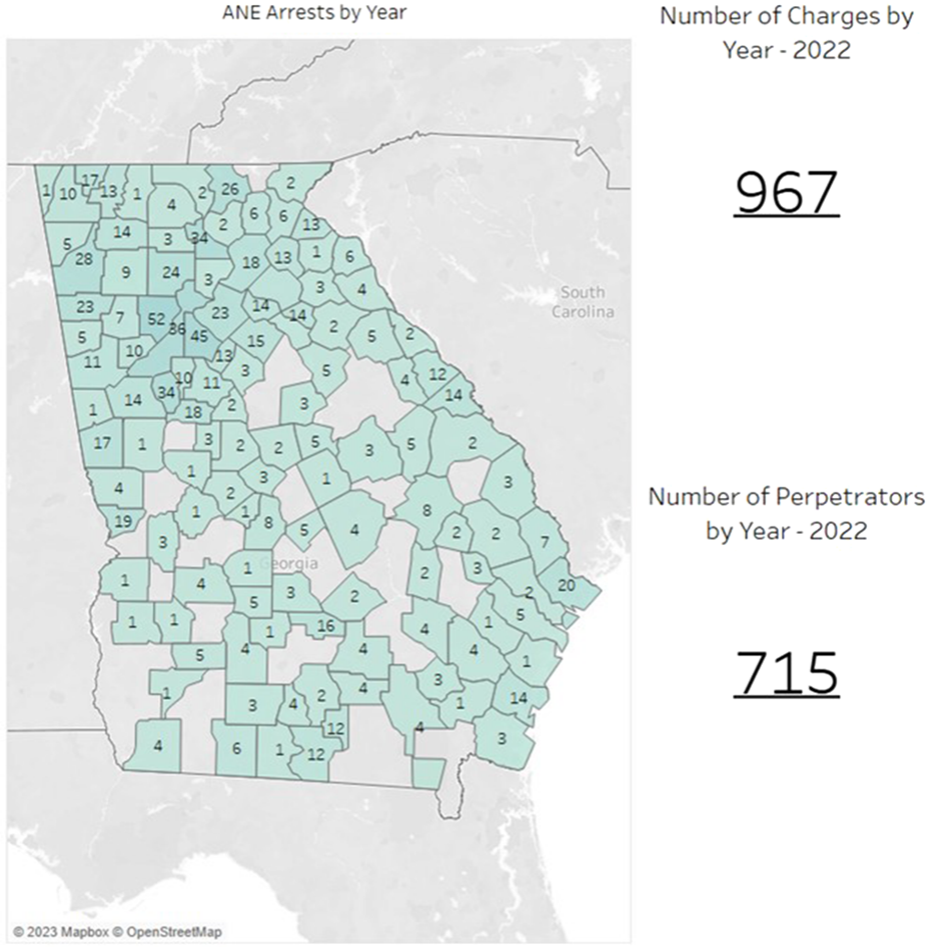


In Georgia, a small network of agencies and people focus on elder abuse and Benefits Trafficking. The Georgia Bureau of Investigation (GBI) is one of these agencies. The GBI collects data relevant to the criminal charges taken by Georgia's law enforcement agencies on statutes related to ANE. This data has been collected and analyzed since 2010 and shows the number of individual criminal warrants taken and the number of people arrested for these criminal charges. These numbers do not include any direct indictments taken by the District Attorney's Offices in each of the judicial circuits. A 230% increase was noted in the number of criminal warrants taken from 2010 to 2015 with 378 criminal warrants in 2010 and 1,251 in 2015 (11). However, during the height of the COVID-19 pandemic in 2020, criminal arrest numbers dropped to 946 warrants, or a −24% decrease from 2015. Since 2020, the percentage has increased by no more than 2% with 967 criminal charges taken. While the number of criminal charges has either decreased or only risen slightly, the number of actual people arrested for at-risk adult ANE criminal charges continues to increase each year. From 2010 to 2015, the number of people arrested increased by 133% from 223 people arrested to 250 people arrested. This number then increased again by 19% between 2015 and 2020, when the number of people arrested was reported as 520 people and 623 people, respectively. By 2022 that number had increased another 14% to 715 people being arrested From 2010 through the end of 2022, the number of people arrested for criminal charges increased by 220% for the 12-year period (Figure 1).

## Georgia benefits trafficking and criminal charges

In 2018, Georgia passed a law against Benefits Trafficking, i.e., trafficking a person for their benefits, O.C.G.A. § 16-5-102.1, “A person commits the offense of trafficking a disabled adult, elder person, or resident when such person, through deception, coercion, exploitation, or isolation, knowingly recruits, harbors, transports, provides, or obtains by any means a disabled adult, elder person, or resident for the purpose of appropriating the resources of such disabled adult, elder person, or resident for one's own or another person's benefit” [(12) amended].

O.C.G.A. § 31-7-12.1 (g) is the criminal statute that makes it a crime to operate an unlicensed personal care home (Unlicensed personal care home; civil penalties; neglect *per se* for certain legal claims; declared nuisance dangerous to public health, safety, welfare; criminal sanctions, 2019 amended). The first offense is a misdemeanor, the second offense, after the initial conviction, is a felony, and if the first offense includes ANE it is automatically a felony. According to Georgia Bureau of Investigation data from 2010 to current, 119 criminal charges were secured for 69 individuals who broke this law. Some of these charges compounded amid larger criminal investigations with numerous other charges and some were only for the misdemeanor or felony charge of operating an unlicensed personal care home.

In Georgia in 2018 when O.C.G.A. § 16-5-102.1 Benefits Trafficking went into effect, there was only one criminal charge against one trafficker. That number increased to eight charges for seven traffickers in 2019, and in 2020, the number decreased back to one arrest and charge. These numbers do not include any direct indictments taken by the District Attorneys' Offices. During the COVID-19 pandemic, no regulatory surveys were conducted for unlicensed personal care homes. Therefore, locating any potential homes where Benefits Trafficking occurred was limited due to resources. In 2021, two charges were taken for two people, and in 2022, there were six charges for six traffickers. It is important to note that criminal charges do not adequately capture the prevalence of Benefits Trafficking. While APS and state regulators may identify clusters of unlicensed homes and relocate the victims, law enforcement is not always equipped to fully investigate and arrest the traffickers. Many of Georgia's law enforcement agencies are in rural areas and are limited in their resources to dedicate the time and attention involved in a Benefits Trafficking investigation.

When a law enforcement agency locates a home with multiple victims who are disabled adults and/or older adults, it can be difficult to understand the complexity of the scene while ensuring everyone remains safe and secure. The investigative work group in Georgia created a questionnaire, which is a combination of the information that APS, law enforcement, and Healthcare Facility Regulation Division require to move each agency's work to the next level. Using one questionnaire creates a trauma-informed response for victims. The questionnaire covers how the victims arrived at the location, health-related questions, government benefits assigned, funds located in financial institutions, and the environment in which the victims lived.

The International Association of Chiefs of Police (IACP) published a collection of roll call videos for responding officers about the different types of elder abuse. One of the videos is based on an unlicensed personal care home investigation in DeKalb County, Georgia. This investigation was conducted prior to the Benefits Trafficking law; however, it shows a success story of rescuing two victims after they were lured into a basement, locked inside the basement, and exploited (13).

**Figure 2:**
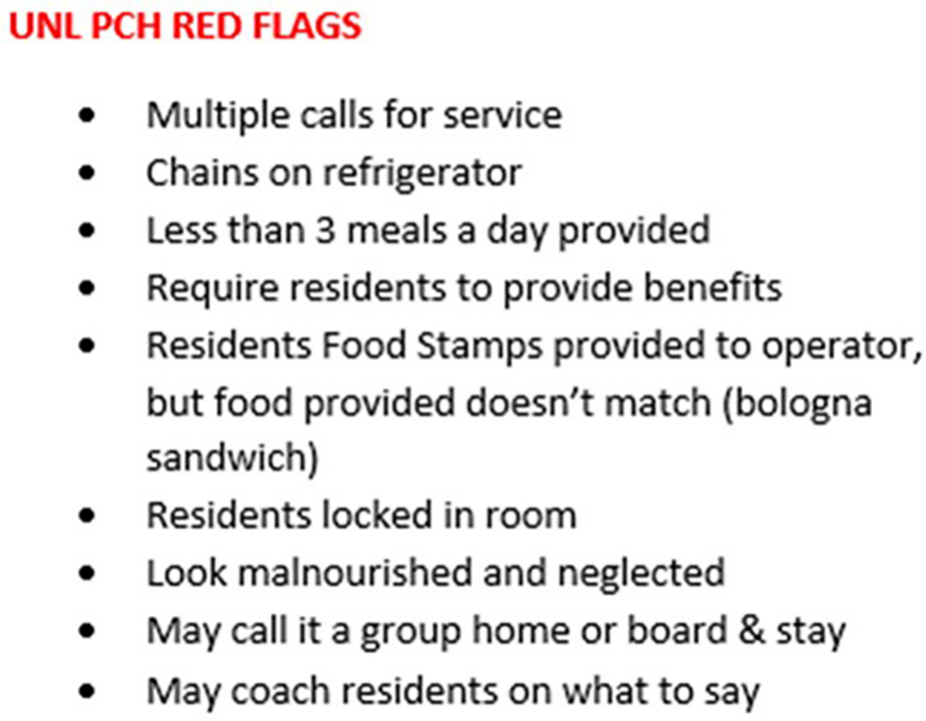


## Conclusion

Implications in awareness of the issue of Benefits Trafficking impacts research, data collection, the legal and criminal justice systems, the public health sector, and at-risk adults themselves. Limitations exist in current data-driven research mainly due to a lack of awareness of the issue and confidentiality laws surrounding APS investigations. There is no central data system to track the victims or the traffickers themselves. Needed data points include how at-risk adult enter the underground system of unlicensed personal care homes, the prevalence of revictimization, the number of homes operated by each individual trafficker, and the prevalence of opening and operating another unlicensed home after being shut down by regulatory agencies and law enforcement. Public health data needed includes what community services the at-risk adults lacked that led them to be easily victimized and how can the lack of certain services be addressed and filled to decrease the need for unlicensed personal care homes. Hospital discharge planners and social workers need training on unlicensed personal care homes and be required to vet the homes they are discharging patients to. The ability to address the abundance of unlicensed personal care homes requires a coordinated community response and agencies with the resources to proactively meet the needs of at-risk adults before they become victims of Benefits Trafficking. Older adults and adults with disabilities often face disadvantages in accessing healthcare, transportation, safe housing, and care and assistance for activities of daily living. Abuse of at-risk adults intersects directly with other social determinants of health and decreases the opportunity for living longer and healthier lives. The cracks in mental health systems, for example, should be filled with funding, staff, and adequate transitional facilities for vulnerable adults to remain in society and get the required care. Without addressing these systemic issues, the crime of Benefits Trafficking will continue to proliferate. Further, criminal justice systems should require training on how to identify, investigate, and prosecute this emerging crime. Benefits Trafficking is nuanced and, like the response needed for victims, criminal justice systems need a coordinated response, funding, and staffing to attack this crime at its root. Moving forward, joint local, state, and federal investigations including data sharing from agencies such as the Social Security Administration and the Veteran's Administration will be necessary to link the network of traffickers and safely relocate and identify victims. Lastly, once Benefits Trafficking is recognized federally as a form of trafficking, research and qualitative data collection funding will hopefully be made available at the state level.

## Supplementary material

Supplementary Image 1 Unlicensed personal care home example 1.

Supplementary Image 2 Unlicensed personal care home example 2.
