# Supplementary figures and images for: Benefits Trafficking: human trafficking of older adults and adults with disabilities
Source: Front Rehabil Sci. 2024 Jan 22;4:1305926. doi: 10.3389/fresc.2023.1305926 (PMC10839064; doi:10.3389/fresc.2023.1305926)

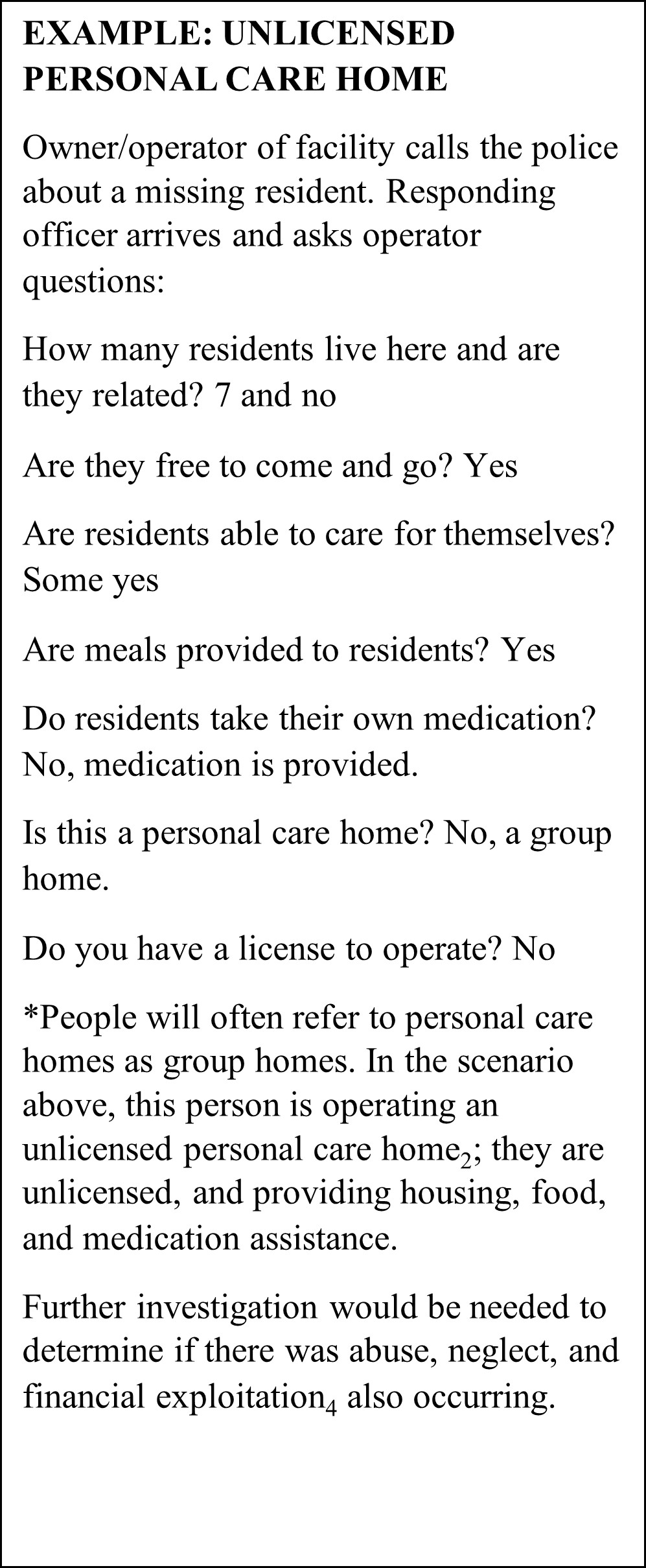

Supplement: Supplementary Image 1 — Unlicensed personal care home example 1. [file Image1.jpeg]

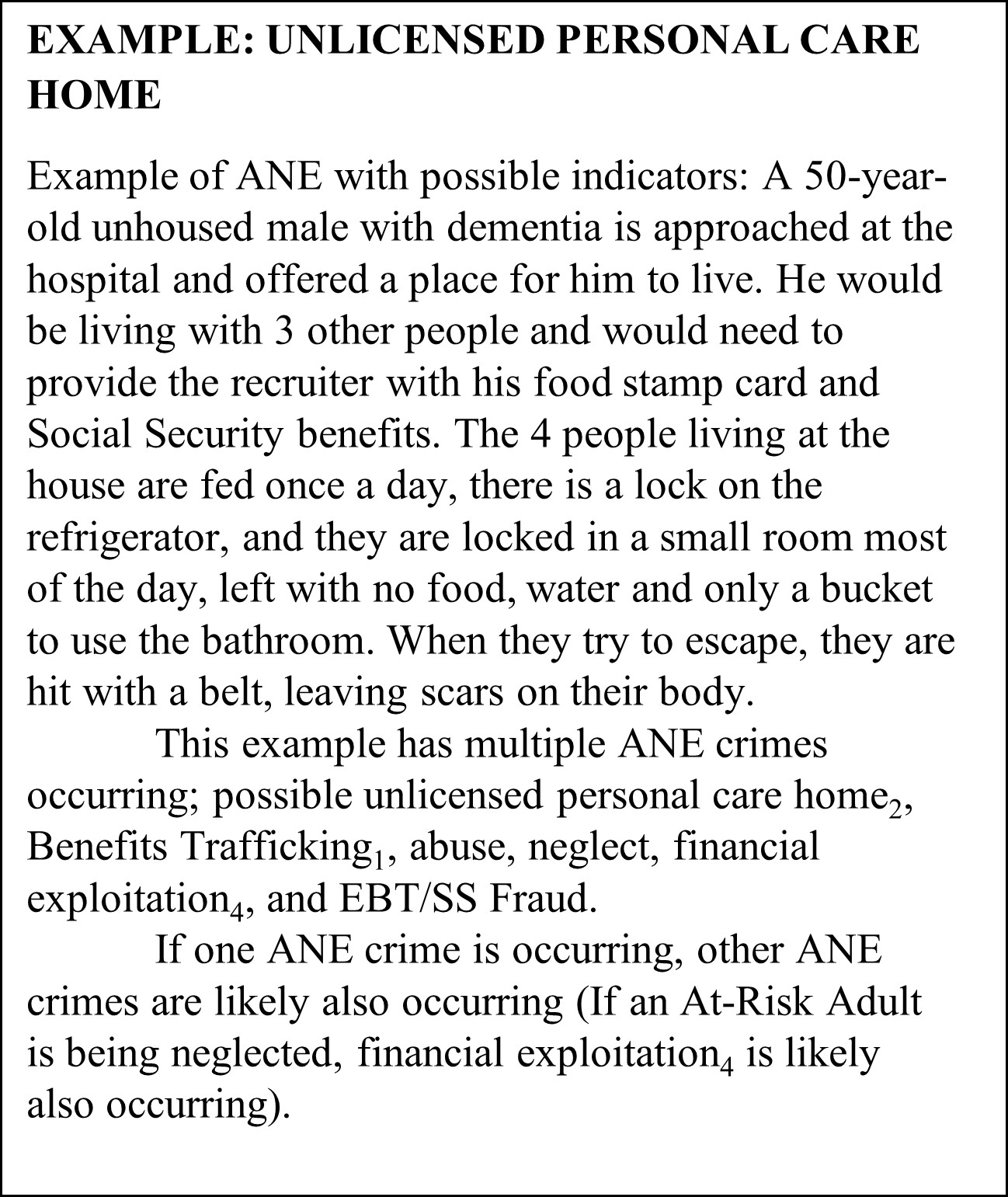

Supplement: Supplementary Image 2 — Unlicensed personal care home example 2. [file Image2.jpeg]
